# Genetic and Molecular Mechanisms of Non-Ischemic Heart Failure with Preserved Ejection Fraction: Pathway Crosstalk, Translational Implications, and Regional Genetic Context

**DOI:** 10.3390/ijms27146203

**Published:** 2026-07-11

**Authors:** Sara Abou Al-Saud

**Affiliations:** Department of Cardiac Sciences, College of Medicine, King Saud University, P.O. Box 7805, Riyadh 11472, Saudi Arabia; saboualsaud@ksu.edu.sa

**Keywords:** HFpEF, non-ischemic heart failure, cardiovascular genetics, consanguinity, inflammation, mitochondrial dysfunction, proteostasis, myocardial fibrosis, epigenetics, biomarkers

## Abstract

Heart failure with preserved ejection fraction (HFpEF) is an increasingly common form of heart failure (HF) that is best understood as a systemic, multiorgan syndrome rather than a disease of left-ventricular filling alone. This review has three specific aims: first, to synthesize genetic and molecular pathways that are most relevant to non-ischemic HFpEF; second, to distinguish HFpEF-enriched mechanisms from evidence extrapolated from ischemic cardiomyopathy or HFrEF; and third, to consider translational implications for populations with high consanguinity, including the Kingdom of Saudi Arabia. The available evidence indicates that chronic inflammatory signaling involving CCL2, CCL5, TLR3, PTGS2/COX-2, IL-6/JAK/STAT3, NF-kB, and NLRP3 acts upstream of endothelial dysfunction, nitric-oxide/cGMP/PKG impairment, mitochondrial reactive oxygen species generation, and fibroblast activation. Extracellular-matrix regulators including ASPN, COL1A1, and MMP2 then amplify collagen deposition and myocardial stiffness, whereas mitochondrial genes and proteins such as ATP5C1 contribute to impaired oxidative phosphorylation, reduced ATP reserve, defective fatty-acid oxidation, and blunted mitophagy. Protein-quality-control pathways involving HSP90AA1, CCT2/CCT5, PSMA3, and stress-responsive STAT3 further link metabolic stress to proteotoxic injury. Epigenetic mechanisms, including DNA methylation and microRNAs such as miR-155, miR-1297, and miR-4649-3p, add a regulatory layer that may improve risk stratification but remains insufficiently validated for routine clinical use. In high-consanguinity settings, recessive cardiomyopathy variants can cluster in families and contribute to earlier NIHF presentations; however, population-level HFpEF-specific variant frequencies remain limited, and findings from HFrEF or dilated cardiomyopathy should be interpreted as candidate pathway evidence rather than definitive HFpEF markers. Translationally, SGLT2 inhibitors, mineralocorticoid-receptor antagonism, biomarker panels, and structured genetic evaluation provide the most clinically actionable bridge from molecular mechanisms to precision HFpEF care.

## 1. Heart Failure (HF) with Preserved Ejection Fraction

**Figure 1:**
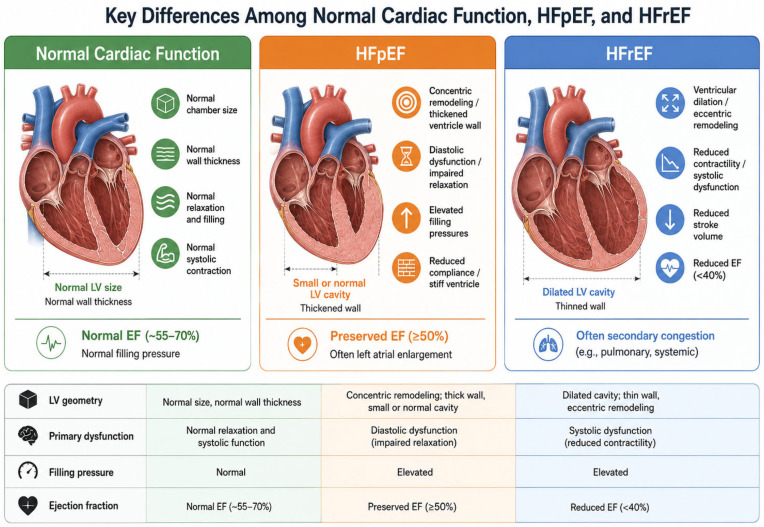
Simplified comparison of normal cardiac function, heart failure with preserved ejection fraction (HFpEF), and heart failure with reduced ejection fraction (HFrEF), highlighting key structural, functional, and pathophysiological differences.

HF is a heterogeneous syndrome characterized by dyspnea or exercise intolerance caused by impairment of ventricular filling, ejection, or both [1]. HF is commonly classified as heart failure with preserved ejection fraction (HFpEF), historically referred to as diastolic HF, and heart failure with reduced ejection fraction (HFrEF). However, this terminology has been debated [2,3]. The American College of Cardiology/American Heart Association guideline framework adopted HFpEF rather than diastolic HF, and the same terminology is used in this review [4]. HFrEF generally reflects impaired systolic pump function and progressive LV dilation when the LVEF is 40% or lower [5]. In contrast, HFpEF is defined by HF symptoms and signs, preserved LVEF, structural or functional cardiac abnormalities, and/or elevated natriuretic peptide levels [6]. HFpEF affects a substantial proportion of adults older than 60 years and accounts for approximately half of HF hospitalizations [7]. Observational data have shown mortality rates comparable with HFrEF in hospitalized cohorts [8], although randomized and prospective studies often show higher cardiovascular mortality in HFrEF [9,10] (Figure 1).

The central purpose of this review is therefore not simply to summarize all HF genetics. Rather, it critically maps molecular pathways that plausibly drive non-ischemic HFpEF, identifies which genes have direct or indirect support in HFpEF biology, and clarifies where data are extrapolated from HFrEF, ischemic cardiomyopathy, or broader non-ischemic cardiomyopathy cohorts. The intended audience includes clinicians who need practical implications for diagnosis, biomarkers, and genetic counseling, and molecular researchers who require a framework of inflammatory, metabolic, proteostatic, epigenetic, and fibrotic signaling networks.

## 2. Pathophysiology of HFpEF

HFpEF is characterized by the signs and symptoms of HF with preserved structural and functional cardiac abnormalities and/or increased natriuretic peptides and with an LVEF greater than 50%, though traditionally HFpEF has been analyzed as a clinical representation of HF in the setting of normal EF [6]. Diastolic dysfunction has appeared as essential to HFpEF disorder, giving a compelling indication of the failure of the Frank-Starling mechanism, stated as the ability to interpret an increase in the filling pressure of the LV to an increase in cardiac efficiency, or only doing so with an abnormally elevated cardiac filling pressure [11]. The main elements of diastolic dysfunction are impaired relaxation of the LV (Decreased Lusitropy) and/or increased LV stiffness (reduced compliance), which frequently coexist, resulting in increased end-diastolic pressure and impaired ventricular filling in the LV pressure–volume loop [12]. But in recent years there has been a paradigm shift from HFpEF as diastolic HF to a more composite multiorgan pattern caused by an interaction of multiple substantial irregularities of the heart that often coexist like LV systolic dysfunction and pulmonary arterial hypertension, chronotropic incompetence/autonomic dysfunction (sympathetic or parasympathetic nervous system), systemic dysfunction of the vascular system (preserved LA reservoir functioning), pericardial limitation, atypical cardiorenal interface, with peripheral abnormalities in terms of skeletal muscles derangements [12,13]. Many of these abnormalities may not be noticed at rest but become apparent under physiological activity or stress conditions with reduced reserve capacity [14,15]. There are certain risk factors for developing HFpEF; the most important of which include hypertension, aging, diabetes, and obesity [11]. A consensus hypothesis among scientists is that the microvascular endothelial inflammatory response is triggered by the systemic multimorbidity-driven pro-inflammatory milieu, leading to microvascular rarefaction and cardiac fibrosis, which synergistically contribute to the development and progression of HFpEF [16]. This inflammatory–fibrotic axis is central to the pathophysiology of HFpEF, distinguishing it from the predominant systolic pump failure of HFrEF, and is driven by specific genetic and molecular pathways discussed in this review. Patients with cardiac issues, LVEF more than 50%, having symptoms secondary to a recognizable cause of diastolic dysfunction and intolerance to exercise should not be considered as HFpEF but should be stated as mimics of HFpEF or HFpEF of secondary cause (due to valvular heart disease, IHD, HTN, and pEF) [9,17].

This exclusion is particularly important for non-ischemic mimics with specific etiologies, including transthyretin or light-chain amyloidosis, hypertrophic cardiomyopathy, storage diseases, constrictive pericardial disease, and primary valvular disease. These disorders can present with preserved LVEF and elevated filling pressures, but their genetic background, myocardial substrate, and treatment logic differ from comorbidity-driven HFpEF; therefore, they should be analysed separately or clearly labelled as comparator phenotypes rather than merged with primary non-ischemic HFpEF cohorts [9,17].

A non-ischemic HFpEF framework places comorbidity-driven systemic inflammation upstream of several myocardial abnormalities. Endothelial activation reduces nitric-oxide/cGMP/PKG signaling, increasing titin-based cardiomyocyte stiffness and favoring hypertrophy. At the same time, inflammatory cytokines and chemokines activate NF-kB, IL-6/JAK/STAT3, TGF-beta/SMAD, and NLRP3 inflammasome signaling, which promotes fibroblast-to-myofibroblast transition, collagen deposition, mitochondrial reactive oxygen species (ROS), and impaired protein quality control. Thus, inflammation, metabolism, proteostasis, and fibrosis should be interpreted as interacting modules rather than isolated pathways [16,18].

Renal and metabolic disease should be treated as mechanistic drivers rather than background comorbidities. Chronic kidney disease increases volume load, neurohormonal activation, endothelial dysfunction, inflammation, oxidative stress, and mineral-metabolism disturbance, all of which promote microvascular rarefaction, myocardial fibrosis, and impaired relaxation. Obesity and diabetes add adipose-tissue inflammation, insulin resistance, lipotoxicity, advanced-glycation signaling, mitochondrial stress, and impaired nitric-oxide bioavailability. These pathways also create plausible gene–environment interactions, in which variants affecting inflammatory tone, extracellular-matrix turnover, mitochondrial energetics, renal sodium handling, and cardiometabolic regulation may modify HFpEF susceptibility and severity [16,18].

## 3. Epidemiology of HFpEF

HFpEF was first identified as an emerging epidemic around 25 years ago. Today, due to the growth of the aging population, the total number of HF-affected people is going to increase over time [10]. One estimate shows that approximately 64 million people are living with HF globally. The prevalence of HF in developed countries is commonly estimated at 1–2% in the overall population [19,20]. The incidence of HF has stabilized, with a decrease in its prevalence in some populations. However, its alarming trend has still been detected in comparatively young individuals, probably associated with an increase in obesity. The estimated prevalence of HF in the USA, Germany, Belgium, and the UK was found to be 2.5%, 4%, 1.3%, and 1.6%, respectively [21,22,23].

In addition to general cardiac anomalies, a clear shift in HFpEF has also been observed in recent years. Approximately 50% of the patients with HF have HFpEF as a major contributor to mortality. According to the latest data, the age-specific incidence of HF is declining in its patients but less so in HFpEF than in HFrEF. Multimorbidity of HF is common in both types (HFpEF and HFrEF), but severity is more common in the case of HFpEF, comprising five or more major comorbidities in 50% of the patients. The risk of HFpEF increases with an increase in age, while obesity, hypertension, and coronary artery disease are additional risk factors [24]. Likewise, evidence proposes that the number of patients with cardiac anomalies might be linked with the rise in developing countries struggling under the high load of communicable diseases and conditions accompanying an advanced Western style of living. The findings of cardiac patients, together with the death rate of HF, are increasing rapidly, as compared to the previous results, indicating that the HF epidemic has not ended yet [25].

All types of HF are prevalent worldwide, with estimates ranging from 4.7 to 13.3% during the last decade. Stable estimates indicate an increasing trend in HFpEF prevalence compared with HFrEF, ranging between 3.8 and 7.4% and 2.4 and 5.8%, respectively [7]. Gender- and age-specific findings show that systolic dysfunction and HFrEF are more prevalent in men, whereas HFpEF is more common in women [26]. HFpEF is predominantly a disease of older people, but its occurrence in individuals younger than 65 years is increasingly linked to obesity, diabetes, hypertension, kidney disease, and other multimorbid states. Therefore, epidemiological interpretation should distinguish general HF prevalence from the non-ischemic HFpEF phenotype that is driven mainly by metabolic, inflammatory, hypertensive, familial, infiltrative, or restrictive mechanisms rather than obstructive epicardial coronary disease [24,27,28].

## 4. Ischemic Heart Failure (IHF) and Non-Ischemic Heart Failure (NIHF)

IHF is the most frequent form of cardiovascular disease, and it is the leading cause of mortality in both genders globally. With hypertension, IHF is accountable for the largest proportion of the 770,000 recently identified cases of HF each year in the United States. However, a recent study has emphasized distinct sex differences in the epidemiology, pathophysiology, and prognosis of IHF and HF. While great steps have been made in detecting the gender discrepancies that exist, the reasons for these alterations remain largely unsolved and signify a prominent area of constant research and growing knowledge. Despite therapeutic advances in acute myocardial infarction (MI), HF remains an acute, long-term outcome of IHF. In response to acute ischemic illness, females are comparatively protected from apoptosis with less experience of cardiac remodeling than males, resulting in preservation of the size and ejection fraction of the LV. Despite these advantages, women are more likely to develop HF as a complication of acute MI but have a lower risk of sudden cardiac death than men [29].

IHF refers to a condition in which oxygen delivery to the heart decreases due to a disturbance in cardiac function [30], typically attributed to coronary artery disease with one or more atherosclerotic plaques. These obstructive plaques cause less coronary artery flow, leading to myocardial ischemic conditions. Complex pathological phenomena of coronary obstruction cause myocardial ischemia. Imbalance in coronary circulation to fulfill myocardial metabolic demands is determined by coronary microvascular dysfunction, and this also disturbs the regulatory mechanism of blood flow, including ion channels, leading to hypoxia, fibrosis, and cell death. The myocardial role will be disturbed, even with the occurrence of atherosclerotic epicardial plaques. This mechanism represents a general linkage among IHF, microvascular dysfunction, and ultimately HF [31]. Coronary myopathy and associated IHF are the leading causes of HFrEF.

Accordingly, ischemic HF should not be restricted to obstructive epicardial coronary artery disease. Coronary microvascular dysfunction can reduce coronary flow reserve, impair endothelial nitric-oxide signaling, generate repeated low-grade ischemic injury, and contribute to fibrosis and diastolic impairment even without a flow-limiting epicardial lesion. This overlap is relevant because microvascular ischemic disease can produce an HFpEF-like phenotype; however, it remains mechanistically distinct from the non-ischemic HFpEF framework when myocardial ischemia is the dominant initiating process [31].

NIHF covers a broad spectrum of diseases, including reduced cardiac function, ventricular dilatation, cardiac sarcoidosis, idiopathic dilated cardiomyopathy, myocarditis, amyloidosis, hypertrophic cardiomyopathy, valvular heart disease, Chagas disease, and arrhythmogenic right-ventricular cardiomyopathy. These conditions can form myocardial scar and provide a substrate for ventricular arrhythmias [32]. Causes of NIHF include genetic, toxic, infectious, iatrogenic, inflammatory, infiltrative, and metabolic processes [33]. Determination of the primary cause has prognostic and therapeutic implications [34], and NIHF contributes to almost one-third of clinical HF cases [35]. Although much NIHF literature focuses on HFrEF, a clinically important subset presents as or progresses to HFpEF, especially when inflammation, fibrosis, hypertrophy, restrictive physiology, amyloid deposition, or metabolic dysfunction increase myocardial stiffness without marked early decline in LVEF.

For this review, non-ischemic HFpEF refers to HFpEF in which the dominant mechanism is not prior myocardial infarction or obstructive epicardial coronary artery disease. This distinction is essential because ischemic HF often begins with cardiomyocyte necrosis, replacement scar, and systolic pump impairment. In contrast, non-ischemic HFpEF is more commonly enriched for endothelial inflammation, concentric remodeling, mitochondrial energetic limitation, fibroblast activation, interstitial fibrosis, restrictive or infiltrative myocardial disease, and familial or recessive cardiomyopathy. Genes discussed below are therefore labelled as HFpEF-enriched, NIHF-associated, or HFrEF/ischemic-derived when the evidence base requires caution.

## 5. Consanguinity as a Risk Factor for HF in KSA

Consanguinity is the marriage of individuals who share a common ancestor, and it increases the likelihood that offspring will express autosomal recessive traits. Earlier genetic studies showed that about 85 percent of cases of isolated congenital heart disease without a recognizable cause follow multifactorial patterns [36]. The same inbreeding patterns that raise the risk of ventricular septal defect, pulmonary stenosis, atrial septal defect, and other structural malformations in first-cousin unions [37] also increase the likelihood of transmitting recessive variants associated with cardiomyopathies that progress to HF. Of particular relevance to HFpEF, consanguinity can increase the burden of variants in genes that regulate myocardial stiffness (e.g., extracellular-matrix components) and metabolic efficiency, both of which are pivotal for diastolic function. In KSA, consanguinity rates range between 20 and 60 percent [38]. This level of inbreeding increases clustering of pathogenic variants associated with dilated cardiomyopathy, arrhythmogenic cardiomyopathy, and restrictive phenotypes, which often present with preserved ejection fraction. These inherited cardiomyopathies present in similar patterns to other recessive disorders seen in consanguineous families, where recurrence risk is higher among siblings [39] and disease expression tends to appear earlier in life. Reports from Middle Eastern populations show higher cardiomyopathy burdens and earlier onset of HF in families with first-degree cousin marriages [40]. CHD continues to carry significant mortality in childhood [41,42]. Still, the long-term consequence of the same genetic mechanisms in adults is increased susceptibility to cardiomyopathic forms of HF, including those leading to HFpEF through pathways of fibrosis and diastolic impairment.

The regional genetic signal should be interpreted with precision. Population-wide frequencies of autosomal recessive variants that specifically cause HFpEF in Saudi Arabia are not yet available. Still, several studies support a high burden of recessive cardiomyopathy in consanguineous families. In infants of Arab descent from the Eastern Province of Saudi Arabia, 55 consecutive dilated cardiomyopathy cases represented 20% of offspring in 41 families; 46% of families involved first-cousin parents, and segregation analysis best fit a recessive model [43]. More recent exome and autozygosity studies in consanguineous Middle Eastern families with cardiomyopathy identified homozygous variants in TNNI3K, DSP, RBCK1, NRAP, KLHL24, and DSC2, including Saudi families with recurrent NRAP and KLHL24 variants and early-onset cardiomyopathy phenotypes [44]. These data do not prove HFpEF specificity, but they show how consanguinity enriches homozygous cardiomyopathy variants that can manifest along a spectrum from dilated/HFrEF to hypertrophic, restrictive, non-compaction, or diastolic phenotypes. Accordingly, Saudi and Gulf HFpEF cohorts require local WES/panel studies, segregation analysis, variant-level frequency reporting, and comparison with non-consanguineous controls before any variant can be considered a validated regional HFpEF marker.

Thus, the additional consanguinity data strengthen the regional rationale but do not justify overclaiming HFpEF-specific causality. The strongest available evidence currently supports enrichment for congenital heart disease, dilated, arrhythmogenic, restrictive, and early-onset cardiomyopathy phenotypes in consanguineous families; HFpEF-focused studies should report phenotype, inheritance pattern, zygosity, segregation, and variant frequency relative to non-consanguineous controls [43,44].

## 6. Genes Involved in HF and Their Mechanisms

Efforts to clarify the molecular basis of cardiomyopathy in HF have relied on gene-expression profiling, proteomic analysis, and integrative multi-omics studies. Earlier work separated HFrEF into ischemic cardiomyopathy, where chronic hypoxia and scar formation drive mitochondrial dysfunction, and non-ischemic cardiomyopathy, where genetic, inflammatory, and structural pathways predominate [45]. Gene Ontology analyses implicate mitochondrial ATP generation, protein folding, proteasomal turnover, immune signaling, and extracellular-matrix remodeling [45,46]. In HFpEF, these broad pathways are interpreted more narrowly: the most relevant perturbations are those that increase cardiomyocyte stiffness, impair lusitropy and energetic reserve, induce endothelial inflammation, activate fibroblasts, and promote interstitial fibrosis without necessarily causing early ventricular dilation. Therefore, the genes in Table 1 should be read as mechanistic candidates across a continuum of evidence, not as a finalized diagnostic panel for HFpEF.

Interpretation of Table 1. Evidence is strongest for HFpEF when a gene or pathway is directly linked to diastolic dysfunction, myocardial stiffness, microvascular inflammation, mitochondrial energetic deficit, or fibrosis. COL1A1, ASPN, inflammatory chemokines, PTGS2/COX-2, mitochondrial oxidative phosphorylation genes and proteostasis markers fit this HFpEF-enriched biology. By contrast, MMP2, STAT3, HSP90AA1, PSMA3, LMNA, SCN5A, and several chaperone/proteasome genes have substantial evidence from HFrEF, ischemic injury, dilated cardiomyopathy, myocarditis, or generalized NIHF models. These should be presented as candidate pathway nodes requiring HFpEF-specific validation, rather than as established HFpEF genetic markers. At present, no single validated germline marker can diagnose non-ischemic HFpEF; a pathway-based interpretation is more scientifically defensible.

## 7. Genes and Epigenetic Mechanisms in Non-Ischemic HFpEF

**Figure 2:**
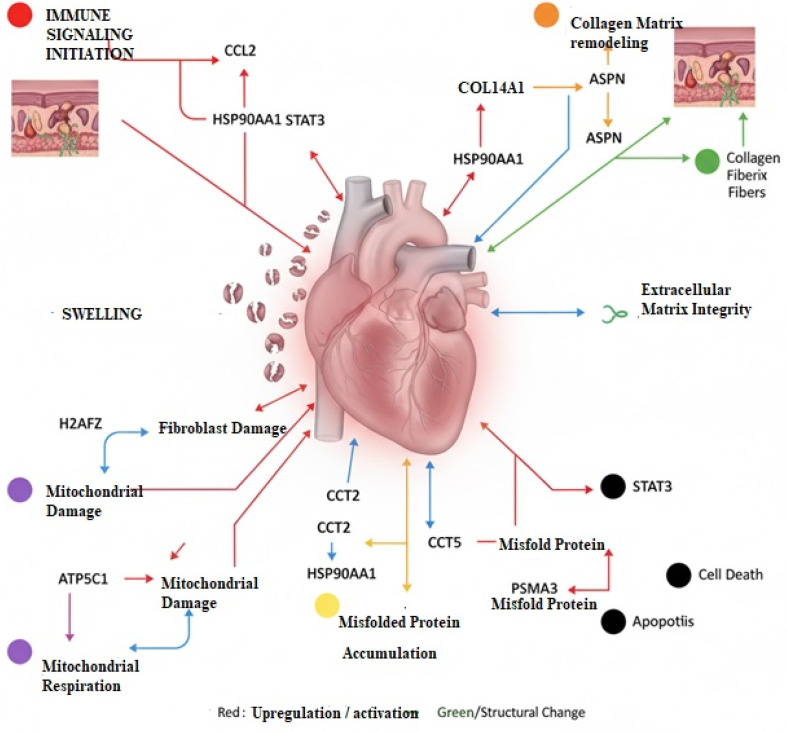
Molecular mechanisms underlying non-ischemic heart failure with preserved ejection fraction. Red arrows/circles: upregulation or activation of inflammatory and stress pathways (e.g., CCL2, CCL5, PTGS2, HSP90AA1, STAT3). Green arrows/circles: structural and extracellular-matrix changes (for example, COL1A1, ASPN) that increase myocardial stiffness. Purple arrows/circles: mitochondrial injury and impaired oxidative phosphorylation (for example, ATP5C1, mitochondrial respiration/ETC dysfunction). Yellow arrows/circles: impaired protein quality control and accumulation of misfolded proteins (e.g., CCT2/CCT5, PSMA3). Black circles: downstream cell death/apoptosis and loss of viable cardiomyocytes. Blue arrows (if present) indicate functional deficits or impaired processes (for example, reduced lusitropy or reduced contractile reserve). Small icons of collagen fibers and fibroblasts indicate extracellular-matrix remodeling and fibrosis. The figure should be interpreted as a pathway-crosstalk model for NIHF/HFpEF, while recognizing that some nodes were first described in HFrEF or ischemic models and require HFpEF-specific validation. Abbreviations used in the figure: ASPN—Asporin; ATP5C1—ATP synthase F1 subunit gamma; CCL2/CCL5—C-C motif chemokine ligands 2 and 5; COL1A1—Collagen type I alpha 1 chain; HSP90AA1—Heat shock protein 90 alpha family class A member 1; PSMA3—Proteasome 20S subunit alpha 3; STAT3—Signal transducer and activator of transcription 3; ETC—Electron transport chain.

HF is a heterogeneous syndrome with diverse causes, pathology, and risk factors [47]. Several studies demonstrate relationships between genetic variation, cardiomyopathy phenotype, arrhythmic risk, and prognosis in NIHF, particularly in familial disease (Figure 2). LMNA and SCN5A variants are important examples because they are strongly linked to arrhythmias and early myocardial dysfunction, with reported major arrhythmic events ranging from 40% to 67% in selected cohorts [48,49]. However, LMNA most classically causes dilated cardiomyopathy and HFrEF; therefore, it is included here as a marker of the NIHF genetic spectrum and arrhythmic risk, not as a validated HFpEF-specific gene. This distinction directly limits extrapolation and avoids implying that a classic HFrEF genotype necessarily causes HFpEF.

Genetic polymorphism analysis can support risk stratification in NIHF but requires phenotype-specific validation for HFpEF. G protein-coupled receptors regulate cardiovascular function, and the beta1-adrenergic receptor Arg389Gly polymorphism suggests that the Gly389 allele is associated with a lower incidence of ventricular arrhythmias in dilated cardiomyopathy cohorts [50,51]. Similarly, the AT1R-1166CC genotype, which affects the renin–angiotensin–aldosterone system, is observed at approximately double the frequency in NIHF patients compared with controls [50]. Circulating miRNAs, including miR-155, also modulate inflammatory and arrhythmic biology [51,52]. These findings are clinically relevant to NIHF, but they should not be presented as HFpEF-specific until replicated in cohorts defined by preserved EF, objective elevation of filling pressures, absence of significant ischemic disease, and adequate adjustment for age, sex, obesity, hypertension, diabetes, kidney disease, and atrial fibrillation.

NIHF involves complex genetic and epigenetic dysregulation that contributes to disease pathogenesis. Analysis of gene expression in NIHF patients reveals dysregulation of nine key genes, with eight up-regulated and one down-regulated, reflecting altered myocardial signaling and remodeling. Among these, prostaglandin–endoperoxide synthase 2 (PTGS2), also known as cyclooxygenase-2 (COX-2), and its associated microRNAs, including miR-1297 and miR-4649-3p, are notable. In NIHF, COX-2 and miR-4649 are significantly upregulated, whereas plasma miR-1297 is significantly decreased, and miR-4649-3p demonstrates strong discriminatory potential for identifying affected individuals [53]. These findings highlight the role of specific gene and miRNA dysregulation in the pathogenesis of NIHF while suggesting potential avenues for biomarker-based risk stratification [53].

Functional gene families and pathways can organize the molecular mechanisms underlying NIHF. Inflammatory and immune signaling genes constitute one key category. For example, CCL5 (C-C motif chemokine ligand 5), located on the q-arm of chromosome 17, is a chemokine that mediates immune cell migration to cardiac tissue, contributing to inflammation-driven myocardial remodeling and promoting the development of NIHF [54]. Similarly, TLR3 (Toll-like receptor 3) participates in pathogen recognition and immune activation. Reduced TLR3 activity impairs innate immune responses in the myocardium, increasing susceptibility to cardiac injury and the progression of HF [55,56]. Integrating these findings with previously described dysregulated genes, such as PTGS2 and associated miRNAs, highlights that inflammatory, immune, metabolic, and structural pathways converge to drive the pathogenesis of NIHF. Subdivision into these functional pathways allows a mechanistic understanding of how genetic and epigenetic perturbations contribute to disease progression and potential targets for therapeutic intervention [56]. A critical synthesis for HFpEF is that these converging pathways—chronic inflammation, metabolic shift, proteostatic failure—collectively elevate myocardial stiffness and impair relaxation—the core diastolic defects—rather than primarily reducing contractility.

## 8. Molecular Crosstalk Among Inflammation, Metabolism, Proteostasis, and Fibrosis

The four core pathways in non-ischemic HFpEF form a feed-forward network. Upstream comorbidities such as obesity, hypertension, diabetes, renal dysfunction, aging, and systemic inflammatory disease increase circulating cytokines, chemokines, free fatty acids, and oxidative stress. Endothelial cells respond by increasing adhesion molecules, lowering nitric-oxide bioavailability and reducing cGMP/PKG signaling, thereby increasing titin stiffness and weakening cardiomyocyte relaxation. In parallel, CCL2/CCL5-mediated monocyte recruitment and PTGS2/COX-2 activity intensify local inflammatory tone, while IL-6/JAK/STAT3 and NF-kB act as transcriptional switches that sustain cytokine production, hypertrophy, and fibroblast activation [16,18].

Downstream, mitochondrial ROS and incomplete fatty-acid oxidation link metabolic stress to inflammation. ROS can activate NLRP3 inflammasome signaling and TGF-beta/SMAD pathways, which promote fibroblast-to-myofibroblast transition and collagen I/III deposition. TGF-beta, STAT3, and mechanosensitive fibroblast signaling then upregulate COL1A1 and other extracellular-matrix genes, while MMP/TIMP imbalance alters collagen turnover and stiffness. The same oxidative and inflammatory environment impairs chaperone and proteasomal systems, leading to accumulation of damaged proteins and further mitochondrial dysfunction. Integrated systems biology and myocardial proteomic studies in HFpEF support this convergence by showing immune activation, oxidative stress, downregulated mitochondrial function and reduced energy metabolism in the HFpEF myocardium [44,57].

In vitro and in vivo evidence supports this sequence but also highlights uncertainty. Endothelial cell and fibroblast studies show that inflammatory stimuli can induce the expression of adhesion molecules, fibroblast activation, collagen synthesis, and STAT3/TGF-beta signaling. Animal models of metabolic HFpEF, including obese diabetic and ZSF1-type models, reproduce diastolic dysfunction, LV hypertrophy, fibrosis, and mitochondrial impairment. However, no model fully captures the heterogeneity of human non-ischemic HFpEF. Therefore, the crosstalk model should be considered a mechanistic framework to organize evidence, not as proof that every pathway node is causal in every patient.

## 9. Mitochondrial Dysfunction and Energetic Reserve in Non-Ischemic HFpEF

Mitochondrial dysfunction is central to non-ischemic HFpEF because preserved LVEF does not imply preserved energy reserve. ATP is required not only for contraction but also for active relaxation, calcium reuptake, actin–myosin detachment, and proteostasis. Defects in oxidative phosphorylation, including ATP5C1-related ATP synthase dysfunction, reduce ATP availability and increase the energetic cost of exercise. This helps explain exertional intolerance and the inability of some HFpEF hearts to augment cardiac output during physiological stress [13,14,15].

Metabolic HFpEF models show a shift away from flexible substrate utilization toward inefficient fatty-acid oxidation, mitochondrial ROS generation, and impaired mitophagy. Blunted mitophagy allows damaged mitochondria to persist, which amplifies ROS, NLRP3 activation, inflammatory cytokine production, and fibroblast activation. A 2024 experimental study found that impaired cardiac mitophagy during metabolic stress contributes to HFpEF and that improving fatty-acid oxidation or mitochondrial quality-control pathways can attenuate mitochondrial dysfunction in the model [57]. These findings strengthen the mechanistic link between energy deficit, inflammation, and fibrosis requested by the reviewers.

## 10. Epigenetic Regulation and Non-Coding RNAs

Epigenetic mechanisms provide a layer of regulation between environmental exposure, comorbidities, and genetic susceptibility. DNA methylation, histone modifications, chromatin remodeling, and non-coding RNAs can alter the expression of inflammatory, metabolic, fibroblast, and extracellular-matrix genes without changing the DNA sequence. In HFpEF, such regulation is biologically plausible because obesity, diabetes, aging, hypertension, and renal dysfunction all influence methylation patterns and inflammatory transcriptional programs.

This review identifies PTGS2/COX-2-associated miRNAs, including increased miR-4649-3p and reduced miR-1297 in NIHF, as well as elevated miR-155 in a subset of NIHF patients [51,52,53]. These miRNAs may link immune activation with arrhythmic risk, endothelial dysfunction, and inflammatory remodeling. DNA methylation may also support risk prediction: a model combining five clinical features with 25 CpG methylation loci predicted early HFpEF risk with an AUC of approximately 0.90 in the Framingham Heart Study cohort [58]. However, these tools require external validation in ethnically diverse cohorts, prospective clinical testing, and clear separation of HFpEF from HFrEF before routine clinical use.

## 11. Translational Applications: Therapeutic Targets, Biomarkers, and Genetic Risk Stratification

The immediate clinical value of the pathways summarized here lies in phenotype-guided treatment and risk stratification rather than single-gene therapy. Current HFpEF management emphasizes control of blood pressure, obesity, diabetes, atrial fibrillation, sleep apnea, congestion, kidney disease, and exercise intolerance [18]. Sodium–glucose cotransporter-2 (SGLT2) inhibitors provide the strongest broadly applicable pharmacologic evidence: empagliflozin reduced the combined risk of cardiovascular death or hospitalization for HF in HFpEF, and dapagliflozin reduced the risk of worsening HF or cardiovascular death in HF with mildly reduced or preserved EF [59,60]. Finerenone, a nonsteroidal mineralocorticoid-receptor antagonist, also reduced total worsening HF events and cardiovascular death in patients with LVEF of 40% or higher, while requiring potassium and kidney-function monitoring because of hyperkalemia risk [61].

Molecular translation is also possible through biomarkers. Natriuretic peptides remain essential for diagnosis and prognosis, but they are influenced by obesity, atrial fibrillation, renal dysfunction, and age. Additional markers such as high-sensitivity troponin, soluble ST2, galectin-3, GDF-15, collagen-turnover markers, MMP/TIMP balance, inflammatory chemokines, and miRNA or methylation panels may better capture myocardial injury, fibrosis, inflammation, and epigenetic risk. At present, these biomarkers are best viewed as complementary tools for phenotyping, prognosis, and trial enrichment rather than stand-alone diagnostic replacements.

Genetic risk stratification is most relevant in early-onset HFpEF, familial HF, restrictive/hypertrophic/non-compaction phenotypes, recurrent unexplained cardiomyopathy in siblings, arrhythmias, sudden cardiac death, and regions with high consanguinity. In Saudi Arabia and similar populations, practical implementation should include careful pedigree analysis, cascade screening when pathogenic or likely pathogenic variants are identified, genetic counseling before and after testing, and interpretation in accordance with ACMG/AMP variant classification standards. Variants of uncertain significance should not be used for predictive clinical decisions without segregation or functional support. These limitations explain why the review does not present a definitive HFpEF genetic panel but instead proposes a pathway-based framework that can guide cohort design and future precision-medicine studies.

## 12. Conclusions

HFpEF arises from a combination of genetic susceptibility, systemic inflammation, metabolic stress, mitochondrial energetic deficit, epigenetic regulation, proteostatic failure, and extracellular-matrix remodeling, rather than from a single mechanical defect. Its increasing occurrence, particularly in older adults and females, reflects population aging and the growing burden of obesity, diabetes, hypertension, kidney disease, and multimorbidity. In high-consanguinity areas, including Saudi Arabia, inherited cardiomyopathies and congenital heart defects can appear earlier and cluster in families, emphasizing the importance of genetic counseling, segregation analysis, and locally representative genomic studies.

The revised synthesis clarifies that some genes and pathways are directly relevant to HFpEF biology, whereas others derive mainly from HFrEF, ischemic cardiomyopathy, or broader NIHF cohorts. LMNA and SCN5A, for example, are clinically important NIHF and arrhythmia genes but are not established HFpEF-specific markers. In contrast, inflammatory chemokines, PTGS2/COX-2, mitochondrial oxidative phosphorylation defects, proteostasis pathways, and ECM regulators such as COL1A1 and ASPN map more closely to diastolic dysfunction, myocardial stiffness, and fibrotic remodeling. This evidence hierarchy prevents overinterpretation while preserving the mechanistic value of earlier HF genetics work.

Clinically, the most actionable bridge from mechanism to practice is phenotype-guided therapy, biomarker refinement, and targeted genetic evaluation in selected patients. SGLT2 inhibitors and mineralocorticoid-receptor antagonism demonstrate that pathways related to metabolism, renal-cardiac inflammation, and fibrosis are therapeutically tractable. Still, HFpEF remains heterogeneous, and no single biomarker or gene is sufficient for diagnosis. Future studies should prioritize non-ischemic HFpEF cohorts with standardized phenotyping, ancestry-aware variant interpretation, multi-omics integration, and validation in consanguineous and non-consanguineous populations. This approach offers the clearest route toward earlier detection, better trial enrichment, and more precise treatment of HFpEF. Equally important, future cohort design should separate primary HFpEF from amyloidosis, hypertrophic cardiomyopathy, restrictive/storage disease, major valvular disease, and microvascular ischemic HF so that biologically distinct mimics do not dilute genetic signals.

## Figures and Tables

**Table 1:** Genetic Mechanisms of Non-Ischemic Heart Failure with Preserved Ejection Fraction.

Gene	Mechanism
CCL2	CCL2 is a CC-chemokine that recruits white blood cells to sites of injury. It activates lymphocytes and monocytes and increases their movement into the myocardium. It supports collagen turnover and changes matrix metalloprotein activity. It also encourages angiogenesis and apoptosis. These combined actions promote chronic inflammation, fibrosis, and structural injury that support the development of heart failure.
MMP2	MMP2 is a matrix-degrading enzyme found in the cardiac extracellular matrix. In idiopathic dilated cardiomyopathy, it is strongly expressed and drives abnormal myocardial remodeling. It weakens left ventricular performance and contributes to interstitial fibrosis. In congestive heart failure, it acts as a key mediator of tissue breakdown and progressive stiffening.
COL1A1	COL1A1 encodes type I collagen, the major fibrillar collagen in the heart. Cardiac fibroblasts produce it. When COL1A1 becomes upregulated, excess collagen accumulates in the myocardium. This causes interstitial fibrosis and leads to stiff ventricles and impaired filling, which are characteristic features of HFpEF.
STAT3	STAT3 supports adaptive signaling in the heart under stress conditions such as ischemia and mechanical strain. It increases anti-apoptotic proteins like Bcl-xL and Hsp70. It also works inside mitochondria to regulate energy production. STAT3 boosts antioxidant enzymes such as MnSOD and reduces oxidative injury. When these pathways become dysregulated, they promote adverse remodeling and end-stage heart failure. Its role in promoting fibrosis and hypertrophy is particularly relevant to HFpEF pathogenesis, though much foundational evidence derives from HFrEF models.
H2AFZ	H2AFZ is a histone variant needed for proper nucleosome assembly. It helps control chromatin structure and gene regulation. When its expression falls, genes related to stress and damage become overexpressed. This loss of regulation promotes pathways associated with heart failure.
ASPN	ASPN is linked with extracellular-matrix changes. It appears in regions of interstitial fibrosis in cardiomyopathies. Its activity supports remodeling of the matrix and contributes to stiff, non-compliant myocardium that limits diastolic function, a hallmark of HFpEF.
HSP90AA1	HSP90AA1 encodes a heat shock protein that becomes activated during ischemia and reperfusion injury. It helps stabilize proteins under stress. High expression signals ongoing cellular injury. Persistent activation indicates heavy cardiomyocyte strain and plays a role in the progression of non-ischemic heart failure.
CCT2 and CCT5	CCT2 and CCT5 are chaperone proteins responsible for folding cellular proteins correctly. When their levels fall, misfolded proteins accumulate inside cardiomyocytes. These include oligomeric amyloid intermediates that are toxic to cells. Their buildup contributes to cardiomyocyte dysfunction, cell death, and clinical heart failure.
PSMA3	PSMA3 is part of the proteasome system that removes damaged and misfolded proteins. When PSMA3 is impaired, protein debris gathers inside cardiomyocytes. This loss of protein quality control promotes apoptosis and remodeling. It is linked with several human cardiomyopathies and supports the progression of heart failure.
ATP5C1	ATP5C1 helps form ATP synthase, the enzyme responsible for oxidative phosphorylation. When ATP5C1 is dysfunctional, ATP production decreases and mitochondrial efficiency drops. Reduced ATP supply weakens the myocardium and limits its ability to handle stress or exercise. This contributes to the energetic deficit and impaired lusitropy seen in HFpEF.

## Data Availability

The original contributions presented in this study are included in the article.

## Abbreviations

CHF	Congestive Heart Failure
DCM	Dilated Cardiomyopathy
HF	Heart Failure
HFpEF	Heart Failure with preserved Ejection Fraction
HFrEF	Heart Failure with reduced Ejection Fraction
IHF	Ischemic Heart Failure
LA	Left Atrium
LV	Left Ventricle
LVEF	Left Ventricular Ejection Fraction
NIHF	Non-Ischemic Heart Failure
NPs	Natriuretic Peptides
ASPN	Asporin
ATP5C1	ATP Synthase F1 Subunit Gamma
AT1R	Angiotensin II Receptor Type 1
Bcl-xL	B-cell lymphoma-extra-large
CCL2	C-C Motif Chemokine Ligand 2
CCL5	C-C Motif Chemokine Ligand 5
CCT2	Chaperonin Containing TCP1 Subunit 2
CCT5	Chaperonin Containing TCP1 Subunit 5
COX-2	Cyclooxygenase-2
COL1A1	Collagen Type I Alpha 1 Chain
GPCRs	G protein-coupled receptors
H2AFZ	H2A Histone Family Member Z
HSP90AA1	Heat Shock Protein 90 Alpha Family Class A Member 1
Hsp70	Heat Shock Protein 70
LMNA	Lamin A/C
miR	microRNA
MMP2	Matrix Metalloproteinase 2
MnSOD	Manganese Superoxide Dismutase
ACMG/AMP	American College of Medical Genetics and Genomics/Association for Molecular Pathology
GDF-15	Growth Differentiation Factor 15
MRA	Mineralocorticoid-Receptor Antagonist
NF-kB	Nuclear Factor kappa B
NLRP3	NOD-, LRR- and Pyrin Domain-Containing Protein 3
NO	Nitric Oxide
PKG	Protein Kinase G
RAAS	Renin–Angiotensin–Aldosterone System
ROS	Reactive Oxygen Species
SGLT2	Sodium–Glucose Cotransporter 2
TGF-beta	Transforming Growth Factor beta
TIMP	Tissue Inhibitor of Metalloproteinases
VUS	Variant of Uncertain Significance
WES	Whole-Exome Sequencing
